# Multiple electrolyte disorders in a neurosurgical patient: solving the rebus

**DOI:** 10.1186/1471-2369-14-140

**Published:** 2013-07-10

**Authors:** Valeria Corradetti, Pasquale Esposito, Teresa Rampino, Marilena Gregorini, Carmelo Libetta, Francesca Bosio, Teresa Valsania, Eleonora Francesca Pattonieri, Chiara Rocca, Stefania Bianzina, Antonio Dal Canton

**Affiliations:** 1 Unit of Nephrology, Dialysis and Transplantation, Fondazione IRCCS Policlinico San Matteo and University of Pavia, Piazzale Golgi 19, 27100 Pavia, Italy; 2 First Department of Anesthesia and Intensive Care, Fondazione IRCCS Policlinico San Matteo and University of Pavia, Pavia, Italy

**Keywords:** Hyponatremia, Polyuria, Hypokalemia, Primary polydipsia, Salt wasting syndromes, Subarachnoid haemorrhage

## Abstract

**Background:**

It is important to ensure an adequate sodium and volume balance in neurosurgical patients in order to avoid the worsening of brain injury.

Indeed, hyponatremia and polyuria, that are frequent in this patient population, are potentially harmful, especially if not promptly recognized.

Differential diagnosis is often challenging, including disorders, which, in view of similar clinical pictures, present very different pathophysiological bases, such as syndrome of inappropriate antidiuresis, cerebral/renal salt wasting syndrome and diabetes insipidus.

**Case presentation:**

Here we present the clinical report of a 67-year-old man with a recent episode of acute subarachnoid haemorrhage, admitted to our ward because of severe hyponatremia, hypokalemia and huge polyuria.

We performed a complete workup to identify the underlying causes of these alterations and found a complex picture of salt wasting syndrome associated to primary polydipsia. The appropriate diagnosis allowed us to correct the patient hydro-electrolyte balance.

**Conclusion:**

The comprehension of the pathophysiological mechanisms is essential to adequately recognize and treat hydro-electrolyte disorders, also solving the most complex clinical problems.

## Background

An optimal balance of sodium and volume is of primary importance in brain-injured patients. In particular, changes in serum sodium concentration (mainly hyponatremia) influence neuronal size, while maintaining an adequate cerebral perfusion pressure avoids further injury [1].

Therefore, hyponatremia and polyuria could represent potentially severe conditions, especially if the underlying causes are not promptly recognized [2].

Differential diagnosis is often challenging, since it includes diseases, which, in view of similar clinical pictures, have very different pathophysiological bases, such as syndrome of inappropriate antidiuresis (SIAD), cerebral/renal salt wasting syndrome (C/RSWS) and diabetes insipidus (DI) [3].

Here we report a case of a neurosurgical patient who presented complex electrolyte disorders associated to marked polyuria.

## Case presentation

A 67-year-old man was admitted in the Infectious Diseases Unit of our hospital because of pneumonia and urosepsis complicating a post-traumatic subarachnoid haemorrhage (SAH).

His past medical history consisted of hypertension, progressive supranuclear palsy-like Parkinsonism and recurrent moderate hypokalemia and slight hyponatremia. His home therapy included angiotensin receptor blockers, angiotensin converting enzyme inhibitors, aldosterone blockers, levodopa/carbidopa and sertraline.

At the admission the patient was febrile and asthenic; blood tests showed severe hyponatremia (Na 120 mEq/l) and hypokalemia (K 2.6 mEq/l), neutrophil leucocytosis, normal liver and renal functions. Multiple antimicrobial therapies, including meropenem and levofloxacin, steroids, tetracosactide and 0.9% NaCl saline infusions were promptly initiated.

In the following days the patient showed negative volume balance (about −2.5 l/day with a median diuresis of 7 l/day), while the arterial blood analysis revealed severe metabolic alkalosis and hypokalemia (pH 7.6, HCO_3_ 46 mEq, Base Excess 21 mEq/l, K 1.5 mEq/l). Simultaneously, his general conditions progressively worsened insomuch as he was transferred to the Intensive Care Unit.

There, the patient was treated with abundant infusions (3–4 l/day) of Ringer’s acetate, KCl i.v. supplementation (60–80 mEq/day) and hypertonic solutions (NaCl 3% at a mean infusion rate of 20 ml/h); blood exams showed Na 124–130 mEq/l, K 2.3 mEq/l and progressive improvement of metabolic alkalosis.

**Figure 1:**
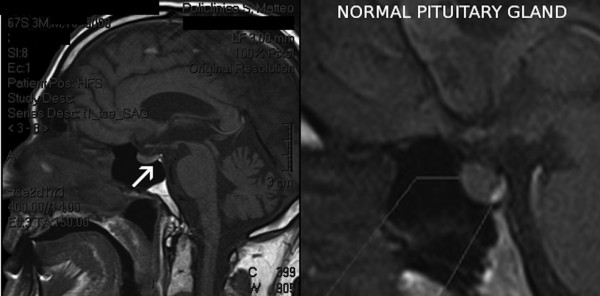
NMR imaging of the turcic sella: arrow shows the absence of the physiological hyper intensity of the neurohypophysis.

Because of the persistent negative liquid balance (−2 l/day) and marked diuresis (6 l/day), insipidus diabetes was suspected and a NMR of the turcic sella was performed. This exam showed an absence of the normal hyperintensity of the neurohypophysis [Figure 1], consistent with the supposed diagnosis; consequently, therapy with desmopressin was started at dose of 2 μg/day i.v. The general and hemodynamic conditions gradually improved, but fluid and electrolyte balance did not change despite desmopressin therapy.

Once stabilised, the patient was transferred to our ward, where he presented hypotension and severely dehydrated oral mucous membranes. With the intent of discerning the spontaneous electrolyte and liquid balance, we stopped desmopressin and steroids administration, beginning a controlled infusion of liquids and electrolytes.

After few days, since the patient still showed persistent polyuria with negative liquid balance (−2/3 l, diuresis 6 l/day), hypokalemia and metabolic alkalosis (pH 7.47, HCO_3_ 30 mEq/l) despite of KCl supplements, we decided to suspend infusions.

The plasmatic osmolarity values ranged from 245 to 292 mOsm/l, urinary osmolarity (Uosm) showed extremely variable levels from 275 to 431 mOsm/l, while ADH (11,9 pg/ml with a normal range < 13), TSH, T4, ACTH and cortisol serum levels resulted normal. Moreover, ultrasonography was negative for renal artery stenosis, as well as for any cardiac alteration.

Our attention was then focused on the Uosm value, suggestive for a mixed diuresis composed of both water and solutes. Urine evaluations revealed a sodium chloride driven diuresis, as proven by the high concentrations of urinary sodium (Na U 900 mEq/24 h with Urinary Anion Gap 40 mEq/l) associated to low urinary glucose and urea levels. This urinary sodium loss was accompanied by an estimated total sodium deficit of about 600 mEq, calculated considering a patient weight of 70 Kg - corresponding to total body water (TBW) of 42 Kg - and an actual natremia of 120 mEq/l [4].

On the basis of these evaluations, together with the evidence of hypovolemia, we diagnosed a salt loosing nephropathy. However, the explanation of the free-water part of the diuresis was still difficult to find. In fact, the patient was no longer receiving i.v. infusions and was confined to bed, reporting to need help to eat and drink.

So, we excluded both iatrogenic excessive water administration and polydipsia.

Aiming to confirm the only missing diagnosis, i.e. diabetes insipidus, supported by NMR findings, we performed a water deprivation test. Surprisingly, we found a preserved urine concentrating capacity (Uosm was 222 mOsm/l at the beginning of the test and 556 mOsm/l at the end). During this test the patient complained of an irresistible desire of water and, unexpectedly, he revealed an ability to drink autonomously a large amount of liquids.

**Figure 2:**
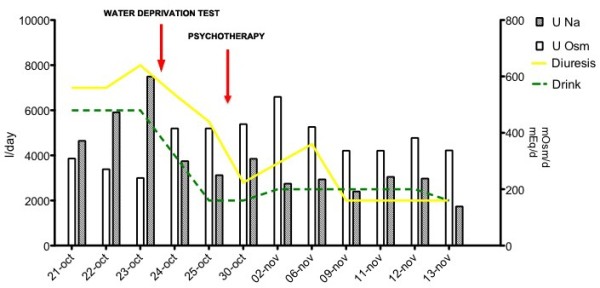
**Time trend of clinical and laboratory parameters during the hospitalization.** Before the water deprivation test the patient declared a daily water intake of about 1 L, but after the beginning of psychotherapy he admitted to have ingested about 6 L of liquids per day. Abbreviations are: Uosm = urinary osmolarity, UNa = urinary sodium.

These findings suggested a diagnosis of primary polydipsia, further confirmed by the effectiveness of psychotherapy, which was associated to a rapid normalization of the volume balance [Figure 2].

Consequently, we prescribed a measured intake of water (2000 ml/day), sodium chloride (19 g/day admi potassium and acid–base equilibrium and well-compensated hemodynamics.

Hence, it was possible to transfer the patient to a rehabilitative hospitalization. Ten months later the patient still presented good general conditions, controlled thirst and water intake, normal serum potassium and sodium levels with only a moderate sodium chloride supplementation (3 g/day).

## Conclusions

The clinical picture of this patient showed the coexistence of many severe and difficult to interpret hydro-electrolyte disorders.

In a patient with a recent history of SAH, the differential diagnosis of hyponatremia comprises erroneous fluid administration, diuretic abuse, acute ACHT deficiency, SIAD, C/RSWS or a combination of these causes [5].

A pathophysiologic approach, considering the assessment of serum and urinary osmolality together with volume status and urinary sodium concentration, may be of help in guiding the diagnostic process (Table 1) [6].

**Table 1:** Differential diagnosis of hyponatremia

**Hyponatremia**						
	**Hypervolemic**	**Euvolemic**			**Hypovolemic**	
**TBW**	++	+	+	+	-	_
**TBNa**	+	N	N	N	-	-
**Urine Na**	-	+	N/+	-	-	-
**Urine Osm**	+	+	+	-	+	+
**Edema**	**+**	**-**	**-**	**-**	**-**	**-**
**BP**	-	N	N	N	-	-
**Diuresis**	-/N	-/N	N	+	_	+
**Possible diagnoses**	CHF	SIAD	Drugs	Primary Polydipsia	NON renal Losses:	Renal Losses:
	Cirrhosis		Nausea			Diuretics
	Nephrotic Syn.		Addison		Vomiting	C/RSWS
					Diarrhea	
					Sweating	

*TBW:* Total Body Water, *TBNa:* Total Body Sodium, *BP:* Blood Pressure, *CHF:* Chronic Heart Failure,

*SIAD:* Syndrome of Inappropriate Antidiuresis, *C/RSWS:* Cerebral Renal Salt Wasting Syndrome. *N:* Normal, +:elevated, -: reduced.

In our case hormone dosing excluded the presence of ACTH deficiency.

Considering that diuretics or excessive amount of fluids were not administrated, the most probably diagnoses remained SIAD or C/RSWS, which differ mainly for the extracellular volume (ECV) status, normal in SIAD while reduced in C/RSWS [7].

SIAD is a disorder of sodium and water characterized by a normovolemic hyponatremia [8]. Since it is mainly a diagnosis of exclusion, all other causes of hyponatremia must be ruled out. The pathophysiological bases are an absolute increase in TBW and impaired urinary dilution ability in absence of any renal disease or stimuli to release ADH [9].

C/RSWS, instead, is a condition of extracellular volume depletion due to a renal sodium transport abnormality with or without high levels of urinary sodium, hyponatremia or cerebral disease [10]. Even if the pathophysiology of the disease is still not perfectly known, some authors hypothesized the presence of abnormal elevations in circulating natriuretic peptides [11,12].

In case of diagnostic uncertainty between SIAD and C/RSWS, the rapid correction of hyponatremia with infusion of saline solutions suggests a diagnosis of hypovolemic hyponatremia [9]. In our case, the presence of hypotension and signs of dehydration, including metabolic alkalosis, together with the evidence of elevated urinary sodium content led us to hypothesize a salt wasting syndrome, which satisfactorily responded to the infusion therapy.

Of note, another factor that potentially contributed to the pathogenesis of hyponatremia was treatment with sertraline, an antidepressant of the selective serotonin reuptake inhibitor (SSRI) class, which use has been already related to hyponatremia, through the development of SIAD [13]. However, since sertraline was stopped soon after the admission, it is unlikely that this drug played a role in the subsequent hydro-electrolyte alterations.

Severe hypokalemia, persistent despite a massive i.v. supplementation, was an additional life-threatening condition that we had to face. Owing to the simultaneous presence of metabolic alkalosis and considering the relationship between K and H ions, it was difficult to determine which was the main culprit.

The high bicarbonate levels could be explained by the severe ECV depletion and the subsequent massive activation of renin angiotensin aldosterone system (RAAS), resulting in the override of the Tmax of proximal tubular bicarbonate reabsorption and the impossibility to excrete the bicarbonate excess [14].

Besides to result from RAAS activation, this marked hypokalemia was probably associated also with the high urinary flux, related to polyuria. However, it has to remark that hypokalemia could itself hold polyuria, causing both increased thirst and mild nephrogenic DI [15].

Actually, the most challenging issue of this clinical case was the explanation of the marked polyuria in such a depleted patient. Polyuria is defined either on a urine flow higher than 3 l/day or on the inappropriateness of the urine flow in relation to ECV or natremia [16]. An easy classification of polyuria could be based on Uosm, which is useful to discriminate between the differences in water and solute diuresis. A Uosm < 150 mOsm/Kg indicates an almost “pure” water-diuresis, a Uosm > 300 mOsm/Kg suggests a “pure” solute diuresis, while a Uosm between 150 and 300 mOsm/Kg (the so-called “mixed” diuresis) needs a closer examination [17]. The possible osmotic role of glucose and urea is easily checked by their quantification in the urine. Instead, in the case of an electrolyte driven diuresis, the measurement of Urinary Anion Gap (UAG: i.e. urinary Na + K − Cl), pH and urinary Na (U Na) are necessary. If UAG < 70 mEq/l and U Na > 250 mmol/24 h, as in our case, a sodium chloride diuresis is confirmed [18].

However, a mixed diuresis is composed also by a free-water part that could be ascribed either to DI or to primary polydipsia [19]. In our case, since imaging bore out the hypothesis of DI, we performed a water deprivation test to confirm this diagnosis. Surprisingly, the deprivation test showed a preserved urine concentration ability, which suggested a diagnosis of primary polydipsia. After an accurate psychological evaluation this condition was attributed to a severe depression caused by the long hospitalization. The fact that psychotherapy rapidly improved patient general conditions provided a further confirmation of our diagnosis.

Interestingly, this condition was also consistent with the contemporary presence of hyponatremia, which, at least in part, could have been caused by polydipsia.

The correct diagnosis was followed by a significant reduction of water intake, thus allowing an adjustment of NaCl supplementation to urinary losses and the achievement of a good hydro-electrolyte balance, which persisted during the time.

In conclusion, the most challenging aspect of this clinical report was the presence of a pool of findings (hyponatremia, hypokalemia and polyuria in a markedly depleted patient), explained by the coexistence of different and often contradictory conditions. An erroneous therapy, as a consequence of an inadequate comprehension of the underlying pathogenetic mechanisms, could have further worsened the already frail clinical setting. A step-by-step and schematic evaluation of all clinical and laboratory signs is necessary to adequately recognize and treat hydro-electrolyte disorders, helping to solve also the most complex clinical problems [20].

## Consent

Written informed consent was obtained from the patient for publication of this Case report and any accompanying images. A copy of the written consent is available for review by the Editor of this journal.

## Abbreviations

C/RSWS: Cerebral/renal salt wasting syndrome; DI: Diabetes insipidus; ECV: Extracellular volume; NMR: Nuclear magnetic resonance; SAH: Subarachnoid haemorrhage; SIAD: Syndrome of inappropriate antidiuresis; RAAS: Renin angiotensin aldosterone system; TBW: Total body water; UAG: Urinary anion gap; U Na: Urinary sodium.

## Competing interests

Authors declare no financial or non-financial interests to disclose.

## Authors’ contributions

VC, PE have been involved in drafting the manuscript analysis and interpretation of data; CR, FB, TV, EFP, SB have made contributions to acquisition of data; MG, TR revising it critically for important intellectual content; ADC has given final approval of the version to be published. All authors read and approved the final manuscript.

## Pre-publication history

The pre-publication history for this paper can be accessed here:

http://www.biomedcentral.com/1471-2369/14/140/prepub

## References

[B1] Ayus JC Arieff AI Pathogenesis and prevention of hyponatremic encephalopathy Endocrinol Metab Clin North Am 1993 22 2 425 446 8325296

[B2] Chawla A Sterns RH Nigwekar SU Cappuccio JD Mortality and serum sodium: do patients die from or with hyponatremia? Clin J Am Soc Nephrol 2011 6 5 960 965 10.2215/CJN.10101110 21441132 PMC3087791

[B3] Schrier RW Body water homeostasis: clinical disorders of urinary dilution and concentration J Am Soc Nephrol 2006 17 1820 1832 10.1681/ASN.2006030240 16738014

[B4] Adrogué HJ Madias NE Hyponatremia N Engl J Med 2000 342 21 1581 1589 10.1056/NEJM200005253422107 10824078

[B5] Sherlock M O’Sullivan E Agha A Incidence and pathophysiology of severe hyponatraemia in neurosurgical patients Postgrad Med J 2009 85 1002 171 175 10.1136/pgmj.2008.072819 19417163

[B6] Thompson C Berl T Tejedor A Johannsson G Differential diagnosis of hyponatraemia Best Pract Res Clin Endocrinol Metab 2012 26 Suppl 1 S7 S15 22469249 10.1016/S1521-690X(12)70003-9

[B7] Upadhyay UM Gormley WB Etiology and management of hyponatremia in neurosurgical patients J Intensive Care Med 2012 27 3 139 144 10.1177/0885066610395489 21345881

[B8] Bartter FC Schwartz WB The syndrome of inappropriate secretion of antidiuretic hormone Am J Med 1967 42 5 790 806 10.1016/0002-9343(67)90096-4 5337379

[B9] Esposito P Piotti G Bianzina S Malul Y Dal Canton A The syndrome of inappropriate antidiuresis: pathophysiology, clinical management and new therapeutic options Nephron Clin Pract 2011 119 1 c62 c73 10.1159/000324653 21677440

[B10] Cerdà-Esteve M Cuadrado-Godia E Chillaron JJ Cerebral salt wasting syndrome: review Eur J Intern Med 2008 19 4 249 254 10.1016/j.ejim.2007.06.019 18471672

[B11] Maesaka JK Imbriano LJ Ali NM Ilamathi E Is it cerebral or renal salt wasting? Kidney Int 2009 76 9 934 938 10.1038/ki.2009.263 19641485

[B12] Yee AH Burns JD Wijdicks EF Cerebral salt wasting: pathophysiology, diagnosis, and treatment Neurosurg Clin N Am 2010 21 2 339 352 10.1016/j.nec.2009.10.011 20380974

[B13] Thornton SL Resch DS SIADH associated with sertraline therapy Am J Psychiatry 1995 152 5 809 7726325

[B14] Sabatini S Kurtzman NA The maintenance of metabolic alkalosis: factors which decrease bicarbonate excretion Kidney Int 1984 25 2 357 361 10.1038/ki.1984.24 6374253

[B15] Weiner ID Wingo CS Hypokalemia–consequences, causes, and correction J Am Soc Nephrol 1997 8 7 1179 1188 9219169 10.1681/ASN.V871179

[B16] Leung AK Robson WL Halperin ML Polyuria in childhood Clin Pediatr (Phila) 1991 30 11 634 640 10.1177/000992289103001104 1747978

[B17] Kamel KS Ethier JH Richardson RM Bear RA Halperin ML Urine electrolytes and osmolality: when and how to use them Am J Nephrol 1990 10 2 89 102 10.1159/000168062 2190469

[B18] Oster JR Singer I Thatte L Grant-Taylor I Diego JM The polyuria of solute diuresis Arch Intern Med 1997 157 7 721 729 10.1001/archinte.1997.00440280015002 9125003

[B19] Edoute Y Davids MR Johnston C Halperin ML An integrative physiological approach to polyuria and hyponatraemia: a ‘double-take’ on the diagnosis and therapy in a patient with schizophrenia QJM 2003 96 7 531 540 10.1093/qjmed/hcg089 12881596

[B20] Laredo S Yuen K Sonnenberg B Halperin ML Coexistence of central diabetes insipidus and salt wasting: the difficulties in diagnosis, changes in natremia, and treatment J Am Soc Nephrol 1996 7 12 2527 2532 8989730 10.1681/ASN.V7122527

